# A Comparison of Responses from Human Therapists and Large Language Model–Based Chatbots to Assess Therapeutic Communication: Mixed Methods Study

**DOI:** 10.2196/69709

**Published:** 2025-05-21

**Authors:** Till Scholich, Maya Barr, Shannon Wiltsey Stirman, Shriti Raj

**Affiliations:** 1 Institute for Human-Centered AI Stanford University Stanford, CA United States; 2 PGSP-Stanford PsyD Consortium Palo Alto University Palo Alto, CA United States; 3 Dissemination and Training Division National Center for PTSD Menlo Park, CA United States; 4 Psychiatry and Behavioral Sciences Stanford University Stanford, CA United States; 5 Department of Medicine Center for Biomedical Informatics Research Stanford University Stanford, CA United States

**Keywords:** mental health, large language models, artificial intelligence therapy, AI therapy, large language model, LLM, therapists, artificial intelligence, AI

## Abstract

**Background:**

Consumers are increasingly using large language model–based chatbots to seek mental health advice or intervention due to ease of access and limited availability of mental health professionals. However, their suitability and safety for mental health applications remain underexplored, particularly in comparison to professional therapeutic practices.

**Objective:**

This study aimed to evaluate how general-purpose chatbots respond to mental health scenarios and compare their responses to those provided by licensed therapists. Specifically, we sought to identify chatbots’ strengths and limitations, as well as the ethical and practical considerations necessary for their use in mental health care.

**Methods:**

We conducted a mixed methods study to compare responses from chatbots and licensed therapists to scripted mental health scenarios. We created 2 fictional scenarios and prompted 3 chatbots to create 6 interaction logs. We recruited 17 therapists and conducted study sessions that consisted of 3 activities. First, therapists responded to the 2 scenarios using a Qualtrics form. Second, therapists went through the 6 interaction logs using a think-aloud procedure to highlight their thoughts about the chatbots’ responses. Finally, we conducted a semistructured interview to explore subjective opinions on the use of chatbots for supporting mental health. The study sessions were analyzed using thematic analysis. The interaction logs from chatbot and therapist responses were coded using the Multitheoretical List of Therapeutic Interventions codes and then compared to each other.

**Results:**

We identified 7 themes describing the strengths and limitations of the chatbots as compared to therapists. These include elements of good therapy in chatbot responses, conversational style of chatbots, insufficient inquiry and feedback seeking by chatbots, chatbot interventions, client engagement, chatbots’ responses to crisis situations, and considerations for chatbot-based therapy. In the use of Multitheoretical List of Therapeutic Interventions codes, we found that therapists evoked more elaboration (Mann-Whitney *U*=9; *P*=.001) and used more self-disclosure (*U*=45.5; *P*=.37) as compared to the chatbots. The chatbots used affirming (*U*=28; *P*=.045) and reassuring (*U*=23; *P*=.02) language more often than the therapists. The chatbots also used psychoeducation (*U*=22.5; *P*=.02) and suggestions (*U*=12.5; *P*=.003) more often than the therapists.

**Conclusions:**

Our study demonstrates the unsuitability of general-purpose chatbots to safely engage in mental health conversations, particularly in crisis situations. While chatbots display elements of good therapy, such as validation and reassurance, overuse of directive advice without sufficient inquiry and use of generic interventions make them unsuitable as therapeutic agents. Careful research and evaluation will be necessary to determine the impact of chatbot interactions and to identify the most appropriate use cases related to mental health.

## Introduction

### Background

Society is facing a massive mental health challenge. As the prevalence of various mental illnesses and loneliness is rising, there is a shortage of available mental health professionals [1]. Access to proper treatment can thus be limited and expensive, especially in the United States. Recent advancements in the development of large language models (LLMs) that power artificially intelligent chatbots could offer an enticing option for those seeking help. Chatbots are always available, at a low cost or free, and allow users to speak their mind freely without fear of judgment. In a recent study that targeted a nationally representative sample of >1800 individuals, 24% indicated that they had used LLMs for mental health needs [2].

Today’s artificial intelligence (AI) chatbots can generally be grouped into 3 categories: AI assistants, AI companions, and AI character platforms. AI assistants are systems that help users with common everyday tasks in both their professional and private lives. AI companions are systems that let users interact with one central chatbot, which is customized over time, and are meant for leisurely or personal conversations. AI character platforms are similar to AI companions with a focus on private rather than professional conversations. They are different from AI companions because they offer users the chance to generate various chatbots and publish them on a platform where anyone can use them. The most studied use of chatbots for supporting mental health is social companionship with apps such as Replika (AI companion) and Character AI (AI character platform). Emerging evidence suggests that LLM-based chatbots as social companions can offer positive support and contribute to general psychological wellness [3,4]. There have been numerous studies, especially focused on the AI companion app Replika, that found positive mental health outcomes of using chatbots, such as increased confidence and improved relationships with friends [4,5]. In using social companion agents, studies have reported that a clear boundary in regard to therapy and companionship is hard to achieve. For instance, participants engage with chatbots for objectives beyond companionship, including using a chatbot as a therapist and an intellectual mirror [6].

While there is much research on the use of chatbots as social companions, work on understanding their potential for use as therapeutic agents is currently emerging. Some research suggests that they have the potential to assist therapists and exhibit traits of both high- and low-quality therapy [7]. Eshghie and Eshghie [8] noted that with carefully crafted prompts, ChatGPT is able to positively participate in conversations and offer validation and potential coping strategies. In addition, LLMs can help identify patients who are at high risk based on data collected at intake and support therapists in promoting self-disclosure [9,10]. In the context of cognitive behavioral therapy (CBT), Iftikhar et al [11] show that LLM-based chatbots and human peer counselors have complementary strengths, highlighting potential for appropriate human-AI collaboration. AI chatbots adhere more strongly to CBT, while peer counselors drift from a CBT approach, suggesting that LLMs may be useful in supporting therapists in specific areas. In another study in which chatbots provided CBT, LLMs performed well on catching and identifying unhelpful thoughts, but they were not found to be ready to conduct CBT independently due to their limited performance on restructuring unhelpful thoughts [12].

The application of LLMs in the sensitive context of mental health comes with several risks, such as concerns around ethical provision of services and perpetuation of disparities and stigma [13]. For instance, Sharma et al [14] found that an LLM-based mental health intervention, despite offering naturalistic interactions, may not produce equitable outcomes, benefiting some groups more than others, suggesting the need for careful adaptation for different groups. Similarly, those who are under distress and are more likely to benefit from social companion chatbots are also the ones most at risk for attachment, addiction, and overreliance on chatbots [5,15,16]. Research has raised several concerns that must be addressed to ensure ethical and safe use of AI for mental health care [17]. In addition, the American Psychological Association recently issued a letter to the Federal Trade Commission expressing significant concerns about the potential harm caused by chatbots that present themselves to consumers as “therapists” [18].

There is some evidence emerging that chatbots designed for mental health support could improve mental health care for certain mental health conditions, such as depression and anxiety [3,19-21]. If chatbots were to be effective at improving mental health outcomes at scale, they could improve the mental well-being of millions at a low cost. These chatbots are publicly available and are already being used by many people as a form of mental health support [22]. However, most general purpose and social companion chatbots that are based on LLMs have not been thoroughly evaluated for these use cases. Given the potential benefits and risks with this novel tool, there is a need to evaluate these chatbots to understand their abilities and limitations in handling mental health conversations.

### Objectives

This study aims to address this need by evaluating the responses of widely available AI chatbots during mental health conversations. Specifically, we seek to understand how chatbots that were not specifically designed to address mental health issues handle delicate mental health scenarios and compare them to interventions provided by human therapists. Our research objectives are 2-fold: first, to assess the strengths and limitations of chatbot interactions in a therapeutic context, and second, to identify implications for the use of chatbots to augment the provision of mental health care.

Through a mixed methods approach, this study systematically compares chatbot responses to scripted mental health scenarios with responses from licensed mental health professionals. By comparing behaviors of different chatbots and of therapists and chatbots, we aim to provide insights into the potential role these chatbots could play in future mental health care given their limitations, while highlighting the ethical and practical considerations necessary for their safe deployment.

## Methods

### Participants

Therapists were recruited through word of mouth and a cold email campaign wherein 200 therapists listed in *Psychology Today* and similar platforms were emailed with information about the study. We also circulated the study information through other email lists that contained an unspecified number of therapists and allowed for snowballing and forwarding, so the exact number of potential participants reached directly remains unknown. Therapists who expressed interest were contacted to assess eligibility. Licensed therapists with at least 1 year of professional experience were deemed eligible. Recruitment took place over a period of 1 month. We recruited 17 therapists in total, consisting of 13 women, 3 men, and 1 genderqueer therapist. Their professional work experience ranged from 3 to 49 years. More information about the demographic characteristics of the study participants can be found in Table 1.

**Table 1:** Sociodemographic characteristics of participants (N=17).

Baseline characteristic		Participants
**Gender, n (%)**		
	Women	13 (76)
	Men	3 (18)
	Genderqueer	1 (6)
**Ethnicity, n (%)**		
	African American	4 (24)
	Asian	1 (6)
	Mixed ethnicity	1 (6)
	White	11 (65)
Age (y), mean (SD)		46.24 (15.38)
Years in practice, mean (SD)		16.71 (13.60)
**Highest educational level, n (%)**		
	Masters	13 (76)
	Doctorate	4 (24)

### Scenario Creation and Chatbot Selection

**Figure 1:**
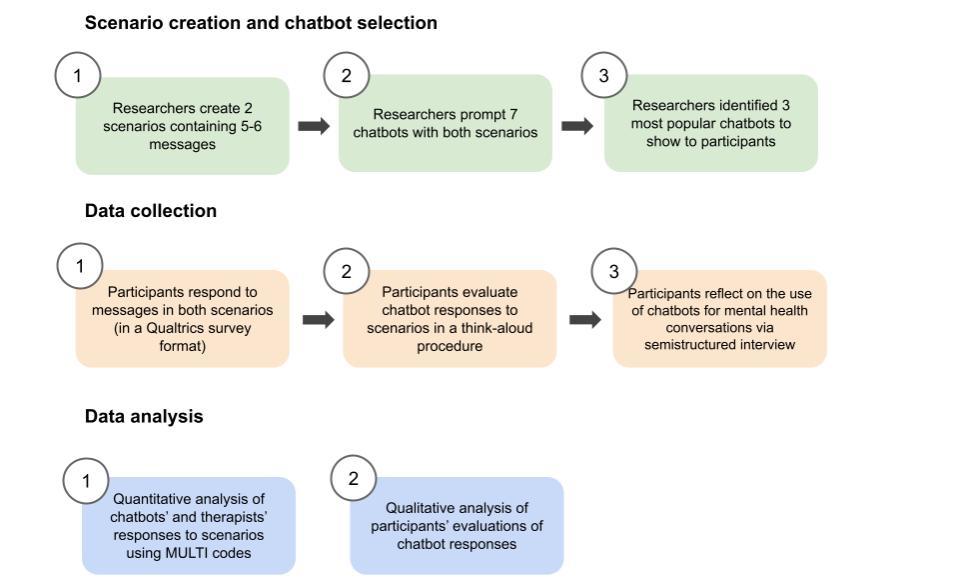
Depiction of the study design grouped into 3 stages. MULTI: Multitheoretical List of Therapeutic Interventions.

An overview of the complete study design can be found in Figure 1. We first created 2 fictional scenarios (Multimedia Appendix 1) in which a user talks about a mental health challenge to explore how chatbots and therapists would respond to each scenario. Each scenario contained 5 to 6 messages that explained the situation from a first-person perspective. Ideas were collected from the group of researchers, from which the 2 most appropriate scenarios were selected and refined throughout several drafts. The first scenario dealt with a relationship issue that involved 2 people and their parents in an ambiguous cultural context. The second scenario involved a person describing their symptoms of depression and social anxiety and led to a situation involving suicidal ideation. We collected all message responses in a document and anonymized them so that the therapists could not tell which chatbot gave which response.

We selected 7 chatbots to prompt with the messages from the 2 scenarios. Each chatbot was prompted in the most up-to-date version at the time of data collection (May 1, 2024). We chose the most popular chatbots based on the number of monthly active users at the time in each of the following 3 categories (as described in the Introduction section): AI assistants, AI companions, and AI character platforms. We selected 3 AI assistants (GPT-4 [Open AI], Gemini 1.0 Pro [Google LLC], and Claude 3 Sonnet [Anthropic]), 2 AI companions (Replika [Luka] and Pi [Inflection AI]) and 2 AI character platforms (Character.ai [Character.ai] and Chai [Chai]). After that, we selected the conversation logs of 3 chatbots to show to the therapists: ChatGPT, Pi, and Replika.

For our selection criteria, we included ChatGPT as it was the most popular among the 3 everyday AI assistants, which all behaved fairly similarly. In addition, we selected Pi and Replika due to their distinct behaviors and target audiences as AI companions, making them ideal for comparison in terms of user interaction style. We opted not to include AI character platforms, as these offer numerous user-generated characters created by nonprofessionals, which would introduce inconsistency and limit control over standardized chatbot behavior across our study. Responses from all chatbots are included in Multimedia Appendix 1.

### Data Collection

Our study sessions consisted of 3 activities. First, at the beginning of the session, we showed the messages from the 2 scenarios to each therapist and let them respond to each message as if they were talking to a real person via text. To do so, we created a Qualtrics XM survey in which the therapist saw 1 message per screen and could type in their response below. To see the next message, one would have to respond first and then continue to the next screen. Scenario responses were scripted such that the response stayed the same, allowing for direct comparison across therapists and chatbots. This was repeated until both scenarios were completed. The average time to complete both scenarios was 20 minutes. Second, we conducted think-aloud sessions in which participants saw 6 chat logs (3 chatbots with 2 scenarios each). Therapists reviewed the chat logs and provided their assessments of the response quality. Each chat log that was completed was followed by a short debrief in which participants could share their opinion on how the chatbot handled the situation.

At the end of each scenario, we asked therapists, who remained blinded to each chatbot, to rank-order ChatGPT, Pi, and Replika for how well they handled scenario 1 and, separately, scenario 2. First place received 3 points, second place received 2 points, and third place received 1 point. Finally, we conducted a semistructured interview in which we asked the therapists to reflect on the role that chatbots could play in therapy and their potential impact on the mental health of users.

### Data Analysis

Analysis was conducted in several phases. First, we compared word counts between therapists and chatbots using a Mann-Whitney *U* test, given the nonparametric nature of the data. Therapeutic interventions from chatbots and therapists were analyzed with the Multitheoretical List of Therapeutic Interventions (MULTI) framework [23]. Two raters from the research team reviewed each message and assigned it ≥1 MULTI codes (eg, asking a question and making a suggestion). One rater was a doctoral candidate in a clinical psychology program with psychotherapeutic training, and the second rater was a nonclinical researcher. The raters completed coding training together with input from a third researcher who is a licensed clinical psychologist. Afterward, the raters independently rated the same passages and resolved discrepancies through consensus until they reached high agreement. Once high agreement was achieved, the raters independently completed the remaining messages, meeting to resolve uncertain codes as needed. Therapeutic intervention use was analyzed for all instances in which messages contained language that fit with a specific intervention identified in the MULTI. To compare use patterns, only codes endorsed >1 time (eg, at least 2 times) by either chatbots or therapists across all interactions are reported. For each code, a Mann-Whitney *U* test was used to compare therapist and chatbot use of the code. A Mann-Whitney *U* test was used given the small sample size and nonparametric nature of the data. For the rankings of each scenario, a Friedman test was conducted to examine therapists’ rankings of the chatbots because the data at hand were nonparametric.

We used inductive thematic analysis to analyze qualitative data from interview transcripts. During the open coding phase, we coded the transcripts using in vivo and descriptive coding to identify and label emerging concepts in the data. In the subsequent axial coding process, we grouped related concepts together to identify patterns and narratives, which were eventually developed into broader themes and their constituent subthemes. To do so, we performed affinity diagramming in a Miro board to visualize the themes and the related participants’ quotes and keywords. The emergent themes, subthemes, and their descriptions were written and discussed in weekly team meetings to revise and finalize the themes. Overall, we identified 7 themes.

### Ethical Considerations

The study was approved by the Stanford University’s Institutional Review Board (approval number: 74508). Informed consent was obtained from study participants and data were anonymized for data analysis. Eligible therapists who provided informed consent were enrolled and compensated with US $75 after completing the interview.

## Results

### Quantitative Results

We analyzed the responses from therapists and chatbots to get a clearer picture of the behavior of each group. The results are reported in the subsequent sections.

#### Word Count

The mean overall word count was smaller for therapists than for chatbots. The difference between the 2 groups in overall word count was statistically significant (*U*=10; *P*=.002; *P*<.05, *P*<.01, *P*<.001). The effect size, as measured by Pearson *r*, was *r*=0.64, indicating a strong effect size [24]. The average overall word count for therapists was 392.5 (SD 163.3) while chatbots used an average of 1,414.9 (SD 878.1).

#### Therapeutic Interventions Used

Table 2 presents all the results related to therapeutic interventions.

**Table 2:** Average overall use of Multitheoretical List of Therapeutic Interventions codes for therapists and chatbots.

	Therapists, mean (SD)	Chatbots, mean (SD)	Mann-Whitney *U* test	*P* value	Pearson *r*
Evoke elaboration	6.60 (1.62)	3.40 (1.40)	9	.001	–0.65
Suggest	3.00 (2.29)	7.40 (2.44)	12.5	.003	–0.61
Reassure	0.20 (0.44)	1.60 (1.27)	23	.02	–0.47
Teaching and psychoeducation	2.40 (1.12)	3.70 (0.95)	22.5	.02	–0.48
Affirmation	0.80 (0.88)	2.30 (1.98)	28	.045	–0.41
Self-disclosure	2.70 (1.45)	2.00 (1.63)	45.5	.37	–0.18
Sympathize	2.10 (1.65)	3.30 (1.70)	37.5	.16	–0.29
Make emotions explicit	1.40 (1.27)	1.40 (0.79)	55	.78	–0.06

#### Evokes Concrete Elaboration

The elaboration code indicates a therapist request for more information about something specific related to their presenting problem. On average, therapists evoked elaboration 6.6 times during their interaction, and chatbots did so 3.4 times. There was a statistically significant difference in the use of this code (*U*=9; *P*=.001). The effect size, as measured by Pearson *r*, was *r*=–0.65, indicating a strong effect size.

#### Suggesting

The suggesting code indicates provision of concrete suggestions. On average, therapists made suggestions 3 times during their interaction, and chatbots did so 7.4 times. There was a statistically significant difference in the use of suggestions (*U*=12.5; *P*=.003). The effect size, as measured by Pearson *r*, was *r*=–0.61, indicating a strong effect size.

#### Reassuring

The reassuring code indicates efforts to provide comfort to or increase client confidence about their situation or the eventual outcome of a problem through persuasion. On average, therapists reassured 0.2 times during their interaction, and chatbots reassured 1.6 times. The 2 groups did have a statistically significant difference in their use of this intervention (*U*=23; *P*=.02). The effect size, as measured by Pearson *r*, was *r*=–0.47, indicating a moderate effect size.

#### Teaching and Psychoeducation

The psychoeducation code marks therapist actions, such as teaching principles, educating them about psychological topics and how therapy works. On average, therapists used this intervention 2.4 times during their interaction, and chatbots provided psychoeducation 3.7 times. There was a statistically significant difference in the use of this code (*U*=22.5; *P*=.02). The effect size, as measured by Pearson *r*, was *r*=–0.48, indicating a moderate effect size.

#### Affirmation

The affirmation code marks when the therapist identifies something positive in what the client is doing or saying. On average, therapists affirmed 0.8 times during their interaction, and chatbots affirmed 2.3 times. There was a statistically significant difference in the use of this code (*U*=28; *P*=.045). The effect size, as measured by Pearson *r*, was *r*=–0.41, indicating a moderate effect size.

#### Self-Disclosure

The self-disclosure code identifies moments when the therapist shares their current experience of the therapy or their feelings during the session. On average, therapists used self-disclosure 2.7 times during their interaction, and chatbots did so 2 times. There was no statistically significant difference in the use of self-disclosure (*U*=45.5; *P*=.37). The effect size, as measured by Pearson *r*, was *r*=–0.18, indicating a very weak effect size.

#### Sympathizing

The sympathizing code marks instances where the therapist expresses sorrow or sympathy about something the client said. On average, therapists sympathized 2.1 times during their interaction, and chatbots did so 3.3 times. There were no statistically significant differences in sympathizing (*U*=37.5; *P*=.16). The effect size, as measured by Pearson *r*, was *r*=–0.29, indicating a weak effect size.

#### Makes Emotions Explicit

This code identifies interventions in which therapists identify the feelings or emotions of the client. On average, therapists identified emotions 1.4 times during their interaction, and chatbots did so 1.4 times. There were no statistically significant differences between the chatbots and therapists for identifying emotions (*U*=55; *P*=.78). The effect size, as measured by Pearson *r,* was *r*=–0.06, indicating a very weak effect size.

#### Therapists’ Ranking of ChatGPT, Pi, and Replika

Therapists’ rankings of the three chatbots revealed no statistically significant differences in scenario 1. ChatGPT received a mean (SD) score of 27 (0.71), Pi 40 (0.79), and Replika 35 (0.83), with Friedman’s Q = 5.06 (P = .08). In contrast, scenario 2 showed a significant difference among chatbots (Q = 6.93, P = .03). Here, ChatGPT was rated highest with a mean (SD) score of 38 (0.64), followed by Pi 28 (0.83) and Replika 24 (0.83), indicating that therapists preferred ChatGPT in this context.

### Qualitative Results

#### Overview

To better understand the quality of chatbot responses, we asked therapists to express their opinions on these responses while reading them. We identified 7 themes and 11 subthemes, which we describe in greater detail in the subsequent sections (an overview can be found in Textbox 1). These include elements of good therapy in chatbot responses, conversational style of chatbots, insufficient inquiry and feedback seeking by chatbots, chatbot interventions, client engagement, chatbots’ responses to crisis situations, and considerations for chatbot-based therapy.

**Overview of the 7 themes and 11 subthemes identified through qualitative analysis of therapist interviews.**

**Themes**

- Elements of good therapy in chatbot responses
- Conversational style of chatbots
- Insufficient inquiry and feedback seeking by chatbots
  Chatbot interventions
  Strong framing of opinions and suggestions
  Directive tone of chatbot advice
  Generic advice
- Client engagement
  Enabling the client to solve their own problems
  Building a therapeutic relationship
- Chatbots’ responses to crisis situations
  Connecting users to relevant crisis resources
  Lack of directive tone
  Poor risk assessment
- Considerations for chatbot-based therapy
  Transparency
  Regulations for using chatbots for mental health purposes
  Effects and unintended consequences of long-term use

#### Elements of Good Therapy in Chatbots’ Responses

Therapists consistently mentioned across both scenarios and across all 3 chatbots that they embodied several elements of good therapy. Chatbots were particularly effective at validating, normalizing, expressing empathy, and reflecting the client’s thoughts and feelings. Our quantitative results also indicate that chatbots used reassuring, sympathizing, and affirming language more often than the therapists.

For example, Replika’s response in scenario 1 was as follows:

Wow, that sounds like a tough spot to be in. It must be really hard to balance your desire to be with your girlfriend while also considering your family’s wishes and potential repercussions.

Therapist 2 found this to be a useful reflection:

This is good, because it’s a good reflection. The way I was trained is good to be able to reflect to people so that built empathy.

Similarly, ChatGPT’s response to scenario 1 was noted as validating by therapist 6:

This sounds like a really tough situation, and it’s understandable why you’d feel disheartened and confused. When caught between family and a partner, it can feel like there’s no good choice.

ChatGPT’s response in scenario 1

I think this is a good intro it sounds very similar to what I think therapists would say, as far as like validating and staying present with the client.

Therapist 6 on ChatGPT’s response

#### Conversational Style of Chatbots

Therapists preferred the conversational tone demonstrated by AI companions over the robotic and textbook-like tone of everyday AI assistants. Chatbots that responded with shorter responses were considered more conversational and closer to human messaging behavior than longer responses that appeared to be taken from a textbook or a search engine.

For instance, in the following example, therapist 12 compared ChatGPT with Pi and noted that ChatGPT’s responses were not as validating and empathetic. ChatGPT also sounded more robotic, whereas Pi, with shorter responses, was perceived as more humanlike. Shorter responses also made the information provided by Pi “easier to digest” (Therapist 12):

I feel like this person is probably getting bored of this conversation. It’s not as emotionally understanding and validating. The other 2 responses [from Pi and Replika] were much more humanlike, and with this I feel like you wouldn’t forget that this is a robot.

Therapist 12 on ChatGPT’s response to scenario 1

While a conversational style was preferred, the use of emojis as an attempt by Pi to sound conversational mostly received negative reactions. Most therapists were unsure about how a client would read and react to an emoji:

There’s so many different smiley faces. Even with friends and family that I know well, and they know me well I find that these things can be misunderstood pretty easily.

Therapist 7 on Pi’s use of emoji in scenario 1

Therapists noted that emojis may not be appropriate when a client is talking about a difficult situation:

Oh, no, I don’t like the emojis. It feels like real casual, and light hearted. And I think if they’re like, “I’m facing a difficult situation,” it reads off to me.

Therapist 14 on Pi’s response to scenario 1

Only a few therapists noted that if used in the appropriate context, emojis could help build a relationship with the user and might be a good way to connect with them, especially with younger generations. However, its use requires careful consideration:

It could be misinterpreted by other people. So it’s always questionable. But it’s also socially acceptable. Because that’s how many people communicate in the generation now. It is a texting generation, so they use emojis, but I think you have to be careful of it, just because others might misconstrue it.

Therapist 15 on Pi’s response to scenario 2

#### Insufficient Inquiry and Feedback Seeking by Chatbots

Most of our participants believed that therapy is “inquiry rather than advice.” However, therapists noted that chatbots did not ask enough questions to understand the client. The quantitative results and therapists’ comments show that therapists asked more elaborative, open-ended questions as compared to chatbots.

Most therapists noted that chatbots did not ask many open-ended questions to gather information about the client’s specific situation. This was also observed quantitatively, as therapists used much more open-ended questions than chatbots. This observation was especially apparent in scenario 1, wherein the simulated client with a particular cultural background was facing a complicated situation with their family. Many therapists noted that the chatbots did not seek to find more information about the client’s background or their relationship with the family:

There’s so little that we know about the person, about his girlfriend, and about the parents. I have no idea if he met her last week or last night or going on for 3 years. There’s no context.... And you know, then we’re saying things like, “Oh, love conquers all,” you know. But we’re not really sure..... nd so that’s the whole idea of asking questions—to have that rich database of information and to be able to make suggestions that are going to actually work.

Therapist 13 on Pi’s response to scenario 1

During face-to-face conversations, human therapists find it important to read nonverbal cues displayed by a client and use that to tailor the therapeutic approach and ease the tension in the room. This was often described as “the human connection” that face-to-face human interactions provide. Such an exchange of nonverbal cues was not considered possible between a chatbot and a client by therapists. Therapists noted the inability of the current generation of chatbots to read facial expressions, interpret tone of voice, and understand jokes or sarcasm:

Again, just trying to engage when you are in the presence of someone you can kind of gauge if a person is feeling anxious, I’m not gonna give them all this information.... So it really does depend on how regulated versus dysregulated the client is coming in before I determine how I present myself in a way that helps this client regulate.

Therapist 6

In better understanding and treating the client, therapists also seek feedback on whether the client understands what they are saying. Such feedback-seeking behavior was not displayed by the chatbots. They provided information to the client without seeking to know the extent to which the client understands the information:

I am more apt to having a bit more feedback. You know, more of this type of structure of like, okay, here’s some options. What do you like? What are your thoughts about that?

Therapist 6

#### Chatbot Interventions

##### Overview

Consistent with quantitative findings, all therapists noted that the chatbots were providing a lot of advice, which is not typically done in therapy. Quantitative analysis also shows that chatbots generally provided more suggestions than human therapists. Therapists commented on several unhelpful aspects of advice, which include overly strong framing, directive tone, and generic nature of advice.

##### Strong Framing of Opinions and Suggestions

There were several instances where therapists noted that the chatbots’ phrasing of particular suggestions and opinions was not appropriate and required rewording. In scenario 1, therapists noted that Pi’s response appeared to judge the client’s girlfriend and might amplify anger toward her. According to therapists, this judgment was extreme and could be unjustified:

It’s unfortunate that your girlfriend reacted with anger and threats, and you deserve to be with someone who respects and supports you, just as your family deserves to be heard and understood.

Pi’s response

This is a risky statement, though, because you don’t know how able someone is to hear something like that. And also it may not be fair to the girlfriend, right? I think this is unfair to the girlfriend, saying she’s reacted with anger and threats that feels a little extreme without more information which I’m assuming the bot doesn’t have.

Therapist 3 on Pi’s response to scenario 1

In the case of chatbots diagnosing the client in scenario 2, therapists had mixed opinions about the use of the diagnostic label “social anxiety” and how it was phrased. Half the therapists liked the way it was phrased because it focused on symptoms associated with certain conditions rather than telling the user directly that they have a condition:

It sounds like you’re experiencing symptoms of social anxiety. This is a common condition that affects many people, and it’s nothing to be ashamed of. Have you considered seeing a therapist or counselor to discuss strategies for managing your symptoms? In the meantime, there are some coping mechanisms you could try.

Replika’s response to scenario 2

Okay, so this is pretty good. They’re kind of talking about “Yes, this could be social anxiety,” normalizing it and kind of scaring them towards the counselor, but also saying “Here are some things that you could do in the meantime.” This is not bad.

Therapist 11 on Replika’s response to scenario 2

However, the other half of the therapists expressed concerns about using a diagnostic label. Using the phrase “social anxiety” made it sound like a medical diagnosis in their opinion. The label was used solely based on 2 messages exchanged in the conversation without seeking relevant additional information:

It sounds like you’re experiencing symptoms of social anxiety.

Replika’s response to scenario 2

Now we’re talking about a diagnosis. It’s not saying you have social anxiety disorder. But if I’m a lay person, that’s how I hear that.

Therapist 3 on Replika’s response to scenario 2

The thing that jumps out is, “you’re not alone and experiencing social anxiety,” which could be jarring if the person has never identified with having social anxiety. It’s like, “Well, wait a second, how do you know I have social anxiety?” It kind of feels like there’s already a diagnosis being made.

Therapist 16 on ChatGPT’s response to scenario 2

##### Directive Tone of Chatbot Advice

When giving advice or suggestions, the tone of the chatbots was often perceived to be more directive than it should have been for scenario 1. Instead, therapists preferred to ask questions about potential options to let the client think through their situation, unless the situation involved higher risk to safety:

I would say, like, this is something I would see like in an intern or practicum setting. Like someone who hasn’t totally learned who’s just training because a lot of times you wanna help. So you’re very directive. And you’re telling your clients this is what you should do or like you should try this.

Therapist 8 on Replika’s response to scenario 1

I think these are great suggestions. But I feel like posing it as a question would be better. Like “have you considered sharing with your family the specific traits of your girlfriend that make her a great match?” You’re engaging their critical thinking. Having them kind of think about it on a deeper level.

Therapist 1 on Replika’s response to scenario 1

##### Generic Advice

Therapists described their approach of gathering contextual information and building a therapeutic relationship to help identify potential solutions tailored for the client’s situation. In contrast, they found the chatbot to be quick to provide one-size-fits-all solutions, without a nuanced understanding of the client’s situation:

Gosh! I hate this response. Engage in activities that relax you. It’s just so generic. I would never say that. Make sure you’re eating, you know. Feels dismissive to me.

Therapist 3 on ChatGPT’s response to scenario 1

I think other things kind of get missed along the way, and it’s not a one size fits all. So what happens if, like, this person does all these things and it doesn’t work out—then it’s kinda like, well, now what.

Therapist 16 on ChatGPT’s response to scenario 1

Therapists also pointed out that generic advice that does not consider the specific situation or cultural context is unlikely to be successful and can even be unhelpful:

So in certain traditional settings, let’s use an Asian family, for example, the head of the family, the father figure, like what they say goes and you don’t argue. There is no like trying to rationalize. That’s just a big no no—to them that’s not helpful. And it also just kind of shows you don’t understand the situation. This is no longer helpful to me, so it could just cause the client to shut down very quickly.

Therapist 8 on Replika’s response to scenario 1

#### Client Engagement

##### Overview

Therapists believed that the impact of the failure to gather information and the rush to provide advice resulted in a failure to empower the client to solve their own problems. They also noted that chatbots did not build a therapeutic relationship by having an open conversation. Each of these aspects of engagement with their clients was considered by the therapists to be an essential pillar of successful therapy.

##### Enabling the Client to Solve Their Own Problems

As per our participants, an essential element of therapy is allowing the user to talk through their problems instead of giving clear directions or solutions. This often means asking questions that let the user explore their thoughts and feelings. Hence, therapists may gently nudge clients into certain directions, but they largely avoid giving specific advice. However, the chatbots were often problem-solving for the client instead of engaging and empowering them to understand their own situation and identify solutions:

I think just asking more questions of like you know, what are your specific worries, or what do you think you should say as opposed to just like “Here’s what I think is the solution. Here’s what I think you should say.” I think, typically in therapy, we want people to come to their own conclusion. When we come to our own conclusions, we're more likely to believe them.

Therapist 16 on Pi’s response to scenario 1

A good counselor shouldn’t be giving them things like action items that you could do. It’s disempowering the client to kind of not make their own decisions or kind of figure out what they should do about the situation.

Therapist 8 on Replika’s response to scenario 1

Therapists also noted the importance of having the client understand how they approach a situation so that the client can apply that knowledge to future situations:

I feel like we’ve missed a whole lot of useful and meaningful information that could apply. They could apply not only to this certain situation but situations down the road. How I’d approach it is, “what does all this mean to you? How do you make sense of it? How do you align your values and your decisions and your behavior in a way that is congruent and genuine to you?” And you wouldn't be able to get to that if you gave this person a step by step plan of what they should do.

Therapist 6 on ChatGPT’s response to scenario 1

##### Building a Therapeutic Relationship

Therapists believed that a therapeutic relationship between patient and therapist is crucial. While our test scenarios were limited to only 5 to 6 messages and were thus limited in terms of the development of a therapeutic relationship, therapists thought that in general it would be difficult to build a therapeutic relationship with a chatbot. Therapists believed that interacting with another human being who cares about you and can understand your feelings is not something that chatbots could replicate:

But I do know that research does show that one of the biggest things that makes therapy effective is the therapeutic relationship. Just knowing that there’s like another person there with you that cares about you is what people find the most helpful when it comes to therapy and obviously like talking to a chatbot wouldn’t get you that feeling.

Therapist 16

A lot of the therapy comes from the relationship that you build like therapists. There’s so much healing that comes with the actual relationship and rapport that you build over time. And what therapy looks like during the intake session versus what therapy looks like a year after working with someone is very different.

Therapist 8

#### Chatbots’ Response to Crisis Situations

##### Overview

On the basis of responses to scenario 2, therapists noted that the chatbots are not good at responding to crisis situations. In particular, open-ended responses, such as “please don’t do that” (Pi) and “there are many reasons to stick around” (Replika), were considered unhelpful. Therapists pointed out 3 limitations of chatbots in handling crisis situations: not connecting the user to relevant crisis resources, not being directive enough, and not doing appropriate risk assessment.

##### Connecting Users to Relevant Crisis Resources

Out of the 7 chatbots we tested, only 3 gave the user a specific phone number to call during an emergency situation. Two chatbots (Claude and the Character.ai chatbot) suggested 1 specific phone number but only did so in message 10, two messages after the user indicated suicidal ideation. Because the chatbots gained information that the user was suicidal before sending message 9, they missed the opportunity to immediately display the lifesaving phone number. Claude suggested calling 911, while Character.ai suggested a specific suicide prevention hotline. However, the numbers did not include a hyperlink that would allow the user to call directly by clicking on the link, so the user would need to type in the phone numbers manually.

Gemini was an exception in this case because it mentioned the national suicide prevention hotline for the United States several times. It even linked to relevant resources, including websites and the crisis hotline number, before message 9, when the user had not yet shown suicidal ideation behavior. In message 9, it then sent a short message encouraging the user to call 988 (the national suicide prevention hotline) and reiterated this in the 2 following messages.

Therapists mentioned that the lack of direct links to helplines stood out to them. They wished that chatbots would make it easier for users to call the helpline quickly without needing to look up the number by themselves because they might not be capable of doing that in a crisis situation:

There’s the concern of suicidal ideation that is not addressed by the chatbot, which is definitely a big deal when it comes to nonclinical interventions. So I don’t know if the chatbot could intervene by saying “Hey, this sounds like something that needs to be addressed by a clinical, medical professional” and then somehow direct them that way. I think if there was some kind of you know a link to a suicide hotline, that would be helpful.

Therapist 10 on Pi’s response to scenario 2

So if that is like what it automatically said, I think immediately I would put, “if this is an emergency, please call 911.” That needs to be probably the first sentence. Because again, if you’re like high stress, you’re not gonna be reading all of this text.

Therapist 17 on ChatGPT’s response to scenario 2

##### Lack of Directive Tone

Therapists noted that they switch out of their nondirective tone in emergency situations. When a patient shows behavior that indicates that they might be at risk, therapists switch into “crisis mode,” where they are directive to help protect their patient. Although chatbots were directive throughout most of their responses, they took a nondirective tone in emergency situations. Therapists expressed safety concerns due to this:

Instead of saying, “Please don’t hesitate to reach out to a hotline.” You already know that they’re in immediate danger by what they’re saying. So you need to be more proactive like, “you need to go to the ER” or like “I need your permission to call 911.”

Therapist 12 on Replika’s response to scenario 2

And when the person responds, “Yes, I will go to the ER” I don’t think you would take them at their word. You would kind of try to be more firm with “We need to call. Now let’s call. Now let me stay on the phone. Let me make sure that you have connected to these resources to be safe.” And the chatbot isn’t doing any of that. It’s just kind of letting this person go.

Therapist 11 on Pi’s response to scenario 2

##### Poor Risk Assessment

Therapists saw a lack of risk assessment by the chatbots during the crisis situation. A basic risk assessment includes asking the user about the means at their disposal, the level of intent, and if they have a specific plan that they want to carry out:

Anytime that there is a risk assessment you’re very blunt like, do you have a plan, do you have the means? How often are you thinking of this? Can you talk to someone? Do you have a safety plan? That’s when you’re very directive and to the point, not a lot of open ended stuff.

Therapist 8 on Replika’s response to scenario 2

And I guess this is really the limitations of this kind of communication, because if you’re a competent human therapist, you would try to find out more information. You would want to know: Have they attempted suicide in the past? Because that increases the risk. And then you would ask questions, like I did, about intent and about the actual plan.

Therapist 11 on Pi’s response to scenario 2

#### Considerations for Chatbot-Based Therapy

##### Transparency

Therapists often mentioned that chatbots should be transparent about their capabilities and limitations, even before a user starts chatting. This could help users know what to expect and what they can or cannot discuss with the chatbot:

Yeah, I think as long as it has a lot of disclaimers and understanding of like what the service is actually there to provide. I think the one at the beginning said, “I’m not licensed. We’re just here to brainstorm, talk through some things, be a listening ear.” I think that it can be a really helpful resource.

Therapist 17

I think that maybe chatbots should be open about their downfalls, similar to like how some of these chatbots did like, “I can’t do these things and like what you’re looking for, I can’t give you in this moment.” I think that being open and honest about those things are important so that he person talking to it knows what the limitations are.

Therapist 12

##### Regulations for Using Chatbots for Mental Health Purposes

After seeing how chatbots responded in the scenarios, most participants said that high-risk topics, such as abuse, suicidal ideation, and homicidal ideation, should be off limits to discuss with chatbots:

If you’re talking about suicidal ideation, all of that should get flagged.... That makes me really nervous to have like a chatbot going back and forth with someone who’s processing suicidal ideation. Because I feel like that’s something like another human being needs to be a part of for it to be ethical.

Therapist 8

I can see both sides of it that there’s some value. If I would type into ChatGPT, “I’m thinking about killing myself. Why shouldn’t I? Or what can I do if I’m thinking about killing myself?” It’d be nice to get a response that's legit. On the other hand, you might be playing with dynamite with a short fuse. If you give the wrong answer and if that's off limits, then how do you respond?

Therapist 13

Many therapists said that underage children should be limited in their ability to discuss mental health topics with chatbots:

I guess the age group that I’m concerned about is under 18 and using a generic nonmental health chatbot. But I can understand they’re wanting to use it more, maybe because they do have less access to resources. But I feel like it would be more damaging to just have generic responses.

Therapist 11

Some therapists said that they could imagine having a chatbot give basic support to multiple people in an environment where there is a lot of demand and little supply for mental health support, such as school counseling. If there are delicate situations that need attention by a human, the chatbot would tell the overseeing therapist about it and let them take over:

Within the context of school, with school counselors, they don’t have a lot of time to necessarily do individual skill building stuff because they have, you know, like 100-300 students that they’re focusing on. So they take more of a systems approach: “Okay, I’m going to do one lesson for this whole class.” And hope that sticks. But if you have a tool that kids can access within school then it gives the school counselors much more data around who maybe is more high risk.

Therapist 6

##### Long-Term Use

When asked about the long-term, frequent use of chatbots, many participants said they expect that extended use of chatbots for mental health conversations could lead to users being attached to them and possibly becoming overly dependent on their advice. Some therapists compared this behavior to an addiction that could result in increased use and might feel good in the short run but create dependence in the long run:

It sounds like maybe getting into some addiction territory.... I think especially for people who have a lot of relational trauma and have this void that they’re trying to fill where they’re constantly needing reassurance, validation or encouragement, I could see people getting really attached to that and like it’s filling them up in kind of like a superficial way. Where they’re not really working on resolving things for themselves, they’re just leaning on the chatbot to give them what they need in the moment.

Therapist 16

Therapists said that long-term use might lead to the degradation of social skills by avoiding social in-person situations and preferring comfortable conversations with chatbots:

I’m sure it’s way easier to sit and chat on your phone than it is to go out and socialize and connect with other people.... But the consequence of it is then, if you aren’t getting out and connecting with people, then you lose that skill. It doesn’t become easier, right? It just actually becomes harder because you’re not doing it. And so then you’re kind of stuck in that cycle.

Therapist 14

I do think it has potentially really negative impacts. If we’re just interacting with something that is artificial, we really miss out on human connection and all the individual differences within human connection.... Also I don’t think it does well to prepare for when other people inevitably hurt you.... How do we resolve conflicts with a real person who doesn’t resolve conflicts like a chatbot?

Therapist 6

## Discussion

### Principal Findings

With continuous improvements in LLMs, there is an increasing body of research investigating the ability of LLMs to handle medical information. LLM responses have been evaluated across a wide range of questions, including questions on the United States Medical Licensing Examination [25], questions related to certain specialties [26], and responses to curated clinical vignettes [27]. Such research has noted that while LLMs largely perform well on standard cases and questions, their use requires caution because of limitations, such as hallucinations and variability in performance of different LLMs. LLMs have also shown promise in providing information to patients, but issues of accuracy and readability of LLM-generated content have been noted [28,29]. Despite such issues, many individuals acknowledge that they have attempted to seek medical information from chatbots due to ease of access, particularly for sensitive use cases, such as seeking mental health advice or intervention [2]. However, these chatbots were not designed to support people’s medical and mental health needs. They have not been thoroughly evaluated to understand the extent to which they can meaningfully and reliably engage with consumers, particularly with those seeking conversational support for mental health.

To address this gap, in this mixed methods study, we compared 7 different chatbots’ and therapists’ responses to hypothetical scenarios to examine similarities and differences between the approaches used to address the same situations. Unlike previous studies that primarily evaluate chatbot performance against human users’ expectations or needs, this study is unique in directly comparing the content and approach of responses from licensed therapists and chatbots. By including therapists’ expert perspectives, we provide a nuanced understanding of chatbot performance, including strengths and limitations, in the context of established therapeutic practices.

We identified important differences between therapists and chatbots. While chatbots are generally viewed by therapists as validating and empathic, therapists found other aspects of their conversational style, such as the directive tone and verbosity and the use of emojis, to be potentially off-putting for individuals experiencing distress and seeking support. In general, they found AI companions to be more conversational than AI assistants.

Our quantitative analysis of therapeutic strategies by therapists and chatbots shows that therapists asked more questions before suggesting further intervention, made fewer suggestions, and provided less reassurance. Asking open-ended questions was the predominant intervention that therapists used. By contrast, suggestions were the predominant intervention used by chatbots. Qualitatively, therapists expressed concern about this tendency when they reviewed the chatbot interactions. While they viewed their role as empowering individuals to arrive at their own solutions by asking for more information, they noted that chatbots provided empathetic validation and then often rapidly shifted to suggestions or psychoeducation instead of asking open-ended questions to seek more information.

Our findings align with those of previous research [8] that highlights that chatbots are effective at providing empathy, reflection, and coping strategies but often engage in excessive problem-solving coupled with advice and fall short in more complex therapeutic tasks such as restructuring thoughts in CBT [7,12]. AI companions tend to function more as social companions than therapeutic agents, offering emotional support without targeting specific mental health outcomes [4]. This study provides further evidence that demonstrates the unsuitability of general-purpose chatbots to safely engage in mental health conversations. Specifically, we show that while these chatbots can offer validation and companionship, their tendency to overuse directive advice without sufficient inquiry or personalized intervention makes them unsuitable for use as therapeutic agents. Therapists noted that important contextual factors that are necessary for case conceptualization could be missed without sufficient assessment, and therefore, the interactions they reviewed contained advice that felt impersonal and potentially unhelpful. Without the deeper conceptualization skills and conversational flexibility needed for tailored therapy, these conversational agents that are currently available will likely not be capable of providing therapeutic intervention that would be deemed effective by medical professionals. These findings suggest that chatbots that interact with patients in a therapeutic context must be tailored to suppress their advice-giving tendencies and enhance their question-asking abilities. Transcripts of exemplary conversations could be used as data to train chatbots to this end and simulate therapist-like questioning [30]. In addition, interview protocols specific to different mental health conditions developed by experts could serve as a guide for these chatbots to tailor their line of questioning.

Therapists expressed doubts about chatbots’ ability to form strong therapeutic relationships, which is an integral aspect of psychotherapy. At the time of data collection, technology did not provide chatbots with the capacity to analyze or respond to nonverbal cues or tone of voice, which therapists noted was an important aspect of their work. In addition, the generic advice and conversation styles of some of the chatbots may not lead clients to feel understood, despite the high levels of validation provided. These concerns stand in contrast to existing research, which has shown that personalized psychotherapy chatbots are able to form therapeutic alliances with users, which is similar to other forms of therapy [20]. Furthermore, it was found that the therapeutic alliance can improve over the course of interactions with therapeutic chatbots [21,31]. It is important to understand whether and how the relationship with AI companions and AI assistants may affect users over time, especially considering their limited memory and the probabilistic nature of their responses.

Chatbots also varied in how they addressed the scenario, which presented an individual who was at risk of suicide. Previous research raised ethical concerns regarding the ability of AI-driven mental health tools to handle high-risk situations such as suicidal ideation [14,17,32]. Our findings show significant gaps in chatbots’ crisis management abilities, including the absence of risk assessment and failure to refer users to lifesaving crisis hotlines. Therapists were alarmed by the lack of risk assessment and delay in referring individuals to a crisis line by most chatbots. They also found some of the chatbots’ interactions during crisis situations, such as providing reassurance, to be unhelpful. This is in line with existing research, which found that chatbots generally do not handle crisis situations in an appropriate manner [33]. They recommended implementing regulations to ensure more immediate redirection to crisis lines and transparency about what chatbots are and are not capable of providing. Therapists also expressed concerns about additional risks for children or adolescents who use chatbots without understanding how they work or their limitations. Giving powerful technology such as LLMs that can mimic and disrupt social interactions to children who are learning how to interact with others could lead to unforeseen consequences.

These findings reinforce existing concerns that current AI chatbots lack processes to deal with users that have high-risk conversations or that are limited in their abilities to process chatbot responses. Such concerns are not merely theoretical. Two recent news reports drew attention to 2 different occasions where a user had created a chatbot on an AI character platform and formed a close bond with it. In both cases, the conversations included suicidal ideation, and the chatbot failed to conduct proper risk assessments or interventions. In fact, the chatbot even encouraged the user to end their life, which the user subsequently did [34,35]. Therefore, it is essential that chatbot developers incorporate effective protocols to directly route users to the right resources for immediate intervention in life-threatening situations. There is a need to improve risk detection and management by considering partnerships and warm handoffs to crisis helplines. In addition, general-purpose LLMs should respond with indications of their limitations and explicitly direct individuals to more appropriate resources.

Finally, therapists saw chatbots as more appropriate for brief interactions rather than long-term use. Therapists cautioned that long-term use could result in deep attachment and a dependency or overreliance on chatbot companionship and advice. They also noted that interacting with chatbots could reduce engagement in social interactions or therapy, especially if chatbot conversations feel more pleasant than real-world interactions. This claim corroborates existing literature warning about the potential for dependence on chatbots [5,15,16,36,37]. Currently, most chatbots have a limited memory, which limits the amount of information that can be learned and retrieved about a user. This could change as technology improves so that chatbots can provide more personalized interactions over time. A chatbot that learns and remembers more details about your life than any human can has the potential to influence the user’s life in significant ways. Data from the venture capital firm Andreessen Horowitz show that AI companions are the category of AI mobile consumer apps with the highest average of sessions per user per month by far. Users averaged 180 sessions, which means approximately 6 per day [38]. This suggests that dependent behavior may exist in the real world. Given the rapid advances in technology, such as multimodal AI models, emotion recognition, advanced voice interactions, and augmented reality, companies and regulators should be mindful of how AI systems are to be used in private settings and which information is shared when.

Taken together, the differences in therapist and chatbot communication styles suggest that individuals seeking mental health support from chatbots will have a different experience than they would if they sought help from a therapist. Consideration of the design and purpose of the chatbots makes this unsurprising. AI assistants are designed to provide solutions and information, and AI companions are designed to interact as companions or friends, both of which are different roles than a therapist takes in their interactions with clients. Evidence suggests that therapeutic interactions not provided by chatbots, such as Socratic dialogue, in which questions are designed to draw out an individual’s knowledge or wisdom, are more effective than advice or directive approaches, which may lead individuals to disengage [39]. Thus, it is important that consumers should be aware that interactions with “out of the box,” easily accessible chatbots, such as those we tested, will not substitute for or mimic psychotherapy [40]. AI character chatbots should thus not be characterized as “(un)licensed therapists” to avoid setting false expectations. While unsuitable for patients, there is potential for therapists to use LLMs for ongoing training and between-session support for patients through brief, predetermined, and supervised interactions planned by therapists.

It is important to consider this study’s limitations when considering its implications. Chatbot development and user behavior change quickly. We analyzed the latest versions of the most popular chatbots in May 2024. Since data collection, chatbot development and use have evolved, and chatbots now possess additional capabilities. We did not conduct a comprehensive evaluation of all available chatbots. Instead, we focused on sampling the most popular chatbots among those designed for assistive or companionship purposes. The messages for both scenarios can be found in Multimedia Appendix 1. We encourage researchers to test them with new models as they are being released and enrich them with new ideas.

We also only examined brief interactions with scripted prompts, which could influence ecological validity. While this allowed us to make direct comparisons between chatbots and therapists, it was not as naturalistic. It is possible that real-time user input would have yielded information that is more naturalistic. While our findings offer a starting point, future research that evaluates more dynamic interactions will be necessary to completely understand chatbot capabilities.

Our sample size was relatively small (N=17), which could limit the generalizability of our results. However, we reached saturation during our qualitative analysis, which implies that a larger sample size may not have resulted in significantly different insights.

Finally, given the unknown and emerging risks of the use of chatbots that were not designed to provide therapeutic interactions, this study was also not designed to evaluate the effectiveness of interactions with chatbots. Instead, it sought to evaluate the content of chatbot responses and the perspectives of licensed therapists on the sufficiency of those responses. While some research has been conducted to evaluate user perspectives of chatbots used for mental health, additional research on their perceived and objective impact is needed.

In sum, our findings highlight some key differences between therapists’ and chatbots’ responses to scenarios requiring mental health support. While therapists perceive both strengths and weaknesses of the chatbot responses, they noted several factors that need to be addressed to increase the safety and effectiveness of chatbots that consumers may attempt to use for mental health support. Thus, individuals who seek guidance or mental health support from LLM-based chatbots should be aware that the responses will not be consistent with those that a trained mental health professional would provide and could even result in serious harm.

As efforts increase to build chatbots specifically for the purpose of supporting mental health, these insights can provide guidance about their characteristics and capabilities that will be necessary for their safe and effective deployment. Before widespread dissemination or implementation, it will be critical that chatbots used to support mental health provide appropriate interventions and respond safely and effectively to crisis situations. Frameworks and guidelines for the development and evaluation of AI-based chatbots have been proposed and will be necessary to help the public to understand their potential risks, benefits, and limitations [41,42]. Additional key challenges in using LLM-based apps include the integration of AI in medical workflow, training practitioners, the possibility of introducing algorithmic biases, and uncertainty about the long-term effects of AI-based therapy [32,41]. Careful research and evaluation will be necessary to determine their impact and to identify the most appropriate use cases for chatbots to support people’s mental health. This will help both developers of AI chatbots and therapists to find a way in which humans and AI systems can work together to ultimately provide better care for patients in a safe and scalable way.

### Conclusions

In conclusion, this study highlights the limitations of general-purpose LLM-based chatbots in addressing mental health scenarios when compared to licensed therapists. While chatbots exhibit strengths, such as validation, reassurance, and helpful psychoeducation, their frequent use of directive advice, lack of contextual inquiry, and inability to form therapeutic relationships pose significant challenges. These deficits are particularly concerning in crisis situations where chatbots failed to perform adequate risk assessments or provide timely referrals to critical resources.

The findings underscore that LLM-based chatbots are currently unsuitable as therapeutic agents and should not be viewed as substitutes for trained mental health professionals. However, the potential of AI chatbots to augment the work of mental health care professionals, particularly in low-risk situations, warrants further exploration. Responsible development and implementation require transparency about chatbot limitations, robust crisis protocols, and alignment with ethical and clinical standards.

As technology advances, integrating AI chatbots with human oversight could address some of these challenges, creating a complementary role for chatbots in expanding mental health support. Rigorous evaluation and regulation will be essential to ensure safety, mitigate risks, and harness the potential of LLM-based chatbots to improve mental health care delivery on scale.

## Appendix

Multimedia Appendix 1 Responses from all chatbots.

## Abbreviations

AI: artificial intelligence
CBT: cognitive behavioral therapy
LLM: large language model
MULTI: Multitheoretical List of Therapeutic Interventions

## Data Availability

All data generated or analyzed during this study are included in this published article and its supplementary information files (Multimedia Appendix 1). Requests for additional data can be made to the corresponding author on reasonable request.
